# Allelic replacement of the streptococcal cysteine protease SpeB in a Δ*srv *mutant background restores biofilm formation

**DOI:** 10.1186/1756-0500-3-281

**Published:** 2010-11-04

**Authors:** Amity L Roberts, Robert C Holder, Sean D Reid

**Affiliations:** 1 Department of Microbiology and Immunology, Wake Forest University School of Medicine, Winston-Salem, North Carolina, 27157, USA

## Abstract

**Background:**

Group A *Streptococcus *(GAS) is a Gram-positive human pathogen that is capable of causing a wide spectrum of human disease. Thus, the organism has evolved to colonize a number of physiologically distinct host sites. One such mechanism to aid colonization is the formation of a biofilm. We have recently shown that inactivation of the streptococcal regulator of virulence (Srv), results in a mutant strain exhibiting a significant reduction in biofilm formation. Unlike the parental strain (MGAS5005), the streptococcal cysteine protease (SpeB) is constitutively produced by the *srv *mutant (MGAS5005Δ*srv*) suggesting Srv contributes to the control of SpeB production. Given that SpeB is a potent protease, we hypothesized that the biofilm deficient phenotype of the *srv *mutant was due to the constitutive production of SpeB. In support of this hypothesis, we have previously demonstrated that treating cultures with E64, a commercially available chemical inhibitor of cysteine proteases, restored the ability of MGAS5005Δ*srv *to form biofilms. Still, it was unclear if the loss of biofilm formation by MGAS5005Δ*srv *was due only to the constitutive production of SpeB or to other changes inherent in the *srv *mutant strain. To address this question, we constructed a Δ*srv*Δ*speB *double mutant through allelic replacement (MGAS5005Δ*srv*Δ*speB*) and tested its ability to form biofilms *in vitro*.

**Findings:**

Allelic replacement of *speB *in the *srv *mutant background restored the ability of this strain to form biofilms under static and continuous flow conditions. Furthermore, addition of purified SpeB to actively growing wild-type cultures significantly inhibited biofilm formation.

**Conclusions:**

The constitutive production of SpeB by the *srv *mutant strain is responsible for the significant reduction of biofilm formation previously observed. The double mutant supports a model by which Srv contributes to biofilm formation and/or dispersal through regulation of *speB*/SpeB.

## Findings

**Figure 1:**
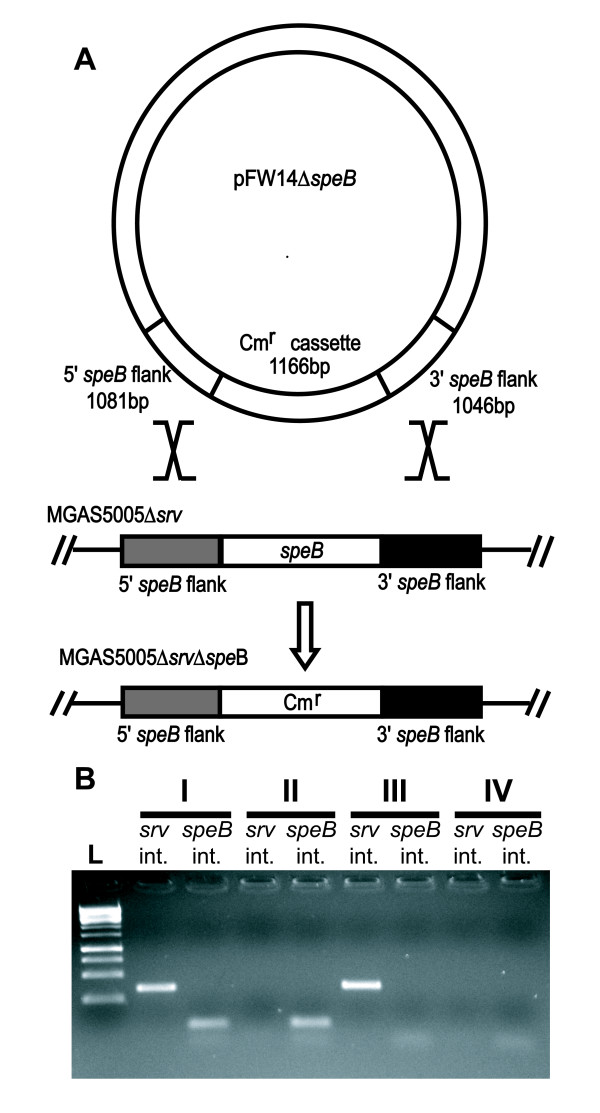
**Construction of MGAS5005Δ*srv*Δ*speB***. (A) *speB *flanking sequences were cloned upstream and downstream of the chloramphenicol resistance cassette *cat *(Cm^r^) in pFW14. The resulting plasmid was transformed into MGAS5005Δ*srv*, and allelic replacement yielded MGAS5005Δ*srv*Δ*speB*. (B) PCR of (I) MGAS5005, (II) MGAS5005Δ*srv*, (III) MGAS5005Δ*speB *and (IV) MGAS5005Δ*srv*Δ*speB *using primers *srv *internal FWD/REV (347 bp fragment) and internal *speB *FWD/REV (80 bp fragment) to verify deletion of the genes *srv *and *speB *within the MGAS5005 mutants. Ladder (L) is a 1 kB ladder.

Group A *Streptococcus *(GAS) is a Gram-positive human pathogen that is capable of causing a wide spectrum of human disease [1-3]. Thus, the organism has evolved to colonize a number of physiologically distinct host sites. One such mechanism to aid colonization is the formation of a biofilm [4-6]. As put forth by Donlan and Costerton, a biofilm is a community of bacteria encased in an extracellular matrix [7]. The structure of this matrix may differ by bacterial species but evidence suggests the biofilm provides protection against the innate host response and therapeutic agents [8-11]. In a study of the biofilm forming ability of 219 clinical GAS isolates representing 32 serotypes, we observed considerable strain to strain variation in biofilm formation based on a crystal violet staining assay (unpublished). This variation has also been observed by others[12]. In our study, one strain named MGAS5005 formed amongst the largest biofilms we observed[13]. MGAS5005 is representative of a M1T1 clone that is globally disseminated and a leading cause of invasive infections world-wide[14-16]. This strain has been shown to have a mutation in the histidine kinase encoded by *covS*, part of the two component regulatory system CovRS (CsrRS)[17]. This mutation results in CovR repression of the cysteine protease* speB*[18,19]. Repression of SpeB is thought to contribute to the invasive phenotype of this clone[17,20,21]. We have recently shown that inactivation of the streptococcal regulator of virulence (Srv), a proposed transcriptional regulator with homology to the *Listeria monocytogenes *regulator PrfA, results in a mutant strain exhibiting a significant reduction in biofilm formation [13,22]. Unlike in the wild-type parental strain, the streptococcal cysteine protease (SpeB) is constitutively produced by the *srv *mutant suggesting Srv contributes to the control of SpeB production [23]. SpeB is capable of cleaving both host (vitronectin, fibronectin, C3b) and self (M-protein, C5a peptidase, Fba, Sda1) extracellular proteins [21,24-30]. Previous studies have shown that SpeB production leads to localized tissue damage and dissemination from the sight of infection in several murine models [31-34]. Given these previous observations, we hypothesized that the biofilm deficient phenotype of the *srv *mutant was due to the constitutive production of SpeB. In support of this hypothesis, we demonstrated that treating cultures with E64, a commercially available chemical inhibitor of cysteine proteases, restored the ability of the *srv *mutant to form biofilms [13]. Furthermore, mature SpeB was undetected in wild-type *in vitro *biofilms by western immunoblot analysis [13]. Still, it was unclear if the loss of biofilm formation by MGAS5005Δ*srv *was due only to the constitutive production of SpeB or to other changes inherent in the *srv *mutant strain. To address this question, we constructed a Δ*srv*Δ*speB *double mutant through allelic replacement (Figure 1). If our hypothesis is correct, biofilm formation would be restored in the MGAS5005Δ*srv*Δ*speB *strain. Furthermore, one would expect that the addition of exogenous SpeB to a growing culture of the wild-type strain would significantly decrease biofilm formation.

The sequence located upstream of the *speB *ORF was amplified from MGAS5005 genomic DNA using *speBsrv *UP FWD (Table 1) and *speBsrv *UP REV (Table 1), generating an ~1.1 kb DNA fragment. The fragment was cloned into the *BsrGI-XhoI *site of pFW14 [22,35,36], forming plasmid pFW14Δ*speB-*UP. Sequence located downstream of the *speB *ORF was amplified from MGAS5005 genomic DNA using *speBsrv *DOWN FWD (Table 1) and *speBsrv *DOWN REV (Table 1), generating an ~1.1 kb DNA fragment. The fragment was cloned into the *XmaI-AgeI *site of pFW14Δ*speB*-UP. The resulting plasmid (pFW14Δ*speB*) was transformed into NovaBlue competent cells (Novagen). Electrocompetent MGAS5005Δ*srv *cells (200 μL) were incubated with pFW14Δ*speB *(2 μg, 10 μL) for 10 minutes on ice. The competent cells and DNA were placed in a pre-chilled 0.2 cm cuvette and electroporated (2.5 kV, 25 μF, 200 Ω). Electroporated cells were incubated for 10 minutes on ice. Cells were allowed to outgrow at 37°C with 5% CO_2 _for 3.5 h in Todd Hewitt broth supplemented with 2% Yeast extract (THY) (Becton, Dickson, Company). Selection for MGAS5005Δ*srv*Δ*speB *occurred on THY agar supplemented with chloramphenicol (5 μg/mL) (Sigma) and incubated at 37°C with 5% CO_2 _for 48 hours. The *speB *deletion was verified in chloramphenicol resistant transformants using PCR and restriction digestion. A PCR utilizing internal *srv *and internal *speB *primers (Table 1) was performed on genomic DNA of MGAS5005 wild-type (I), MGAS5005Δ*srv *(II), MGAS5005Δ*speB *(III) and MGAS5005Δ*srv*Δ*speB *(IV) (Figure 1B) to validate deletion of either *srv *or *speB *or both within the indicated mutants.

**Table 1:** Primers and probes used in this study

Primer or probe	Sequence
*speB *internal FWD	5'-TCAACATGCAGCTACAGGATGTG-3'
*speB *internal REV	5'-TCAACCCTTTGTTAGGGTAATTATGATA-3'
internal *srv *FWD	5'-GCATTGTGAAACAGAGTGTTCTTTCAAAATATGG-3'
internal *srv *REV	5'-TAGTTCTTCGCCAAATAGGGTCATTAAGTC-3'
*prsA *309AA FWD	5'-GCGACAGTCGTGACCTTATCAG-3'
*prsA *309AA REV	5'-CTGACAGTGATGGTGTCTCCTTTC-3
*prsA *309AA Probe	5'-CATCACACAACAACACCAAACTCGTC-3'
*speBsrv *UP FWD	5' ATATATATTGTACACGATAATAGGTTTGCCTAGTGAG-3'
*speBsrv *UP REV	5'-ATATATATCTCGAGGCTAAAAGACTTAATAATCTGACACC-3'
*speBsrv *DOWN FWD	5'-ATATATATCCCGGGCAGTATACTACCAAGGTGTCGG-3'
*speBsrv *DOWN REV	5'-ATATATATACCGGTCGCCAGCGTTACCACTC-3'
*gyrA *FWD	5'-CGACTTGTCTGAACGCCAAA-3'
*gyrA *REV	5'-TTATCACGTTCCAAACCAGTCAA-3'
*gyrA *Probe	5'-CGACGCAAACGCATATCCAAAATAGCTTGE-3'

To verify that *speB *mRNA was not produced by MGAS5005Δ*srv*Δ*speB*, total RNA was isolated from MGAS5005 (control) and MGAS5005Δ*srv*Δ*speB *and subjected to TaqMan real-time reverse transcriptase PCR (RT-PCR) analysis [37,38]. Results indicated that transcript was not produced for either *srv *or *speB *(data not shown) in the MGAS5005Δ*srv*Δ*speB *strain. Transcript of *prsA*, a gene located immediately downstream of *speB*, was ~ 3 fold higher in MGAS5005Δ*srv*Δ*speB *than MGAS5005, indicating that transcription of downstream genes was not disrupted. It should be noted that MGAS5005Δ*srv *[22] and MGAS5005Δ*speB *have previously been shown to be free of detectable polar effects [31,34,39]. Also, Srv and SpeB have both been shown to be produced by MGAS5005 [22,23].

**Figure 2:**
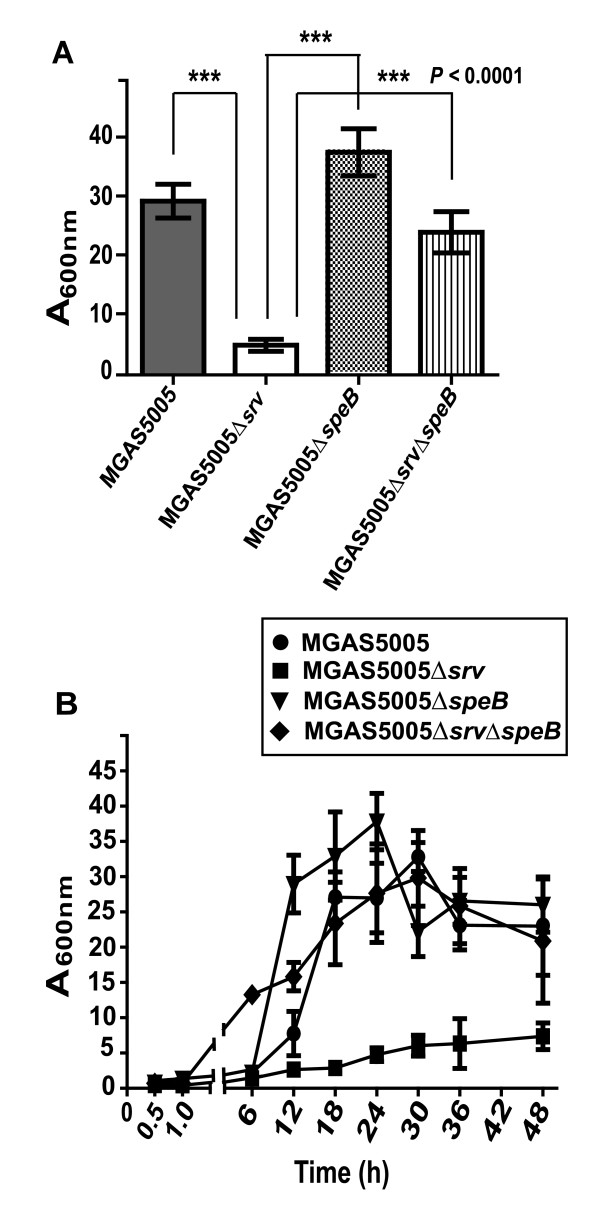
**Static crystal violet assays for the measurement of *in vitro *biofilm formation**. MGAS5005, MGAS5005Δ*srv*, MGAS5005Δ*speB *and MGAS5005Δ*srv*Δ*speB *were grown in 6-well tissue culture treated polystyrene plates for 24 h (A), stained with crystal violet, and solubilized with ethanol. Each reported value for the CV assay is an average of at least 6 replicates and is adjusted by the dilution factor required to obtain a spectrophometric reading (A_600 nm_) (P ≤ 0.0001, unpaired t-test). (B) Biofilm formation for each strain over time is shown out to 48 h.

To examine biofilm formation, MGAS5005, MGAS5005Δ*srv*, MGAS5005Δ*speB *[31,34,39] and MGAS5005Δ*srv*Δ*speB *cultures were grown under static conditions (0.5 h - 48 h); biofilm production was measured through crystal violet (CV) staining as previously described [13] (Figure 2). Inactivation of *speB *in the *srv *mutant background restored biofilm formation to near wild-type levels after 24 h (Figure 2A). Inactivation of *speB *in the MGAS5005 wild-type background does not alter biofilm formation (Figure 2A). MGAS5005Δ*srv*Δ*speB *formed significantly more biofilm than that of MGAS5005Δ*srv *(*P *≤ 0.001, unpaired student's t-test). Over time, biofilm formation of MGAS5005Δ*srv*Δ*speB *closely resembled what we have previously reported for MGAS5005 with maximal formation occurring between 24 h and 30 h with a subtle decline in CV staining thereafter (Figure 2B) [13]. Planktonic growth of MGAS5005, MGAS5005Δ*srv*, MGAS5005Δ*speB*, and MGAS5005Δ*srv*Δ*speB *indicated that there was no growth defect of the mutant strains compared to the wild-type as equivalent bacterial loads were recovered over time (e.g. AVG 8.32 ± 0.72 Log10 CFU/mL 7 h post-growth initiation).

**Figure 3:**
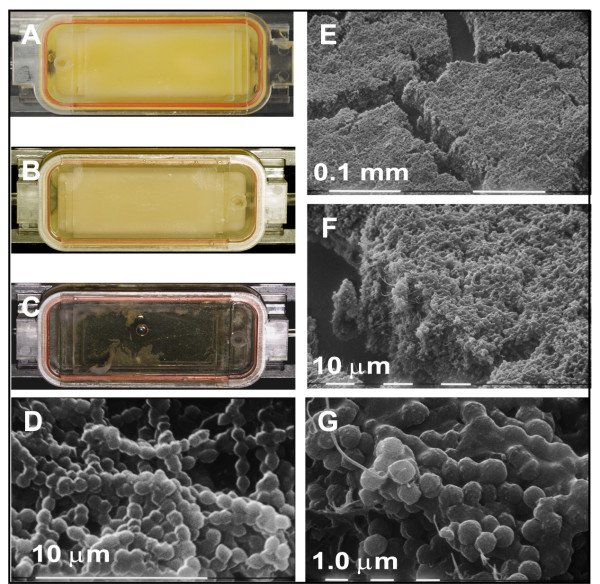
**MGAS5005Δ*srv*Δ*speB *biofilm formation under continuous flow conditions**. (A-C) Representative flow cell chambers containing 24 h grown cultures under a flow rate of ~ 0.7 mL/min of MGAS5005Δ*srv*Δ*speB*, MGAS5005, and MGAS5005Δ*srv*, respectively. (A and B) Chambers inoculated with (A) MGAS5005Δ*srv*Δ*speB *or (B) MGAS5005 were filled with dense viscous material indicative of GAS biofilms. (C) MGAS5005Δ*srv *was unable to form biofilms under flow conditions. Scanning electron microscopy of a 24 h (D) MGAS5005 and (E-G) a MGAS5005Δ*srv*Δ*speB *continuous flow biofilm clearly depicts chains of cocci organized into a 3-dimensional structure encased in a matrix-like material.

Studies have shown that hydrodynamic shear forces are often needed for biofilm formation as these conditions are comparable to that of the host environment [40-42]. MGAS5005Δ*srv *was unable to form a biofilm under continuous flow conditions [13]. To verify that the restored biofilm phenotype observed for MGAS5005Δ*srv*Δ*speB *was maintained under continuous flow, MGAS5005Δ*srv*Δ*speB *was grown in a flow cell chamber under a flow rate of ~ 0.7 mL/min for 24 h as previously described [13]. After 24 h, the flow chamber was completely filled with a viscous substance (Figure 3A) comparable to flow chamber grown wild-type MGAS5005 (Figure 3B). Once again, MGAS5005Δ*srv *failed to attach and form a biofilm under these conditions (Figure 3C). Electron microscopy revealed a dense population of MGAS5005Δ*srv*Δ*speB *organized in a three-dimensional structure (Figure 3E-G). Visually, this structure is equivalent to the MGAS5005 biofilms we have observed (Figure 3D) [13]. Higher magnification revealed chains of MGAS5005Δ*srv*Δ*speB *(Figure 3G) which appeared to be coated in a matrix material comparable to what has been seen in MGAS5005 biofilms (Figure 3D) [13]. Thus, MGAS5005Δ*srv*Δ*speB *can form a biofilm under continuous flow conditions.

**Figure 4:**
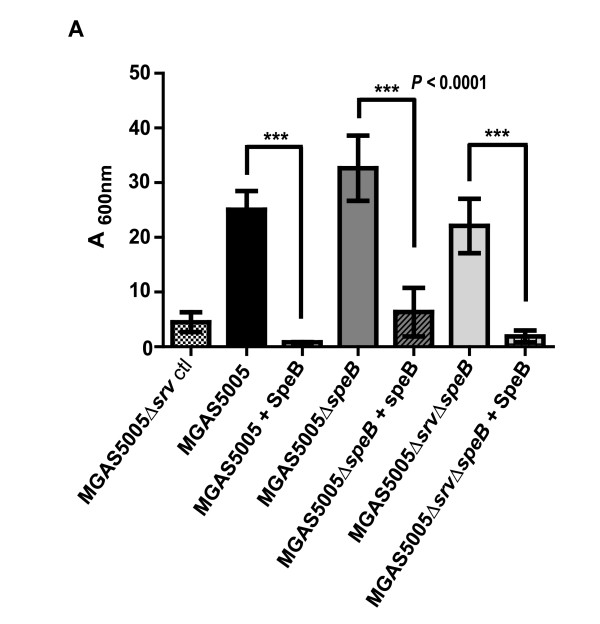
**Addition of purified active SpeB inhibits biofilm formation**. MGAS5005, MGAS5005Δ*speB *and MGAS5005Δ*srv*Δ*speB *were either untreated or treated with 1 μg/mL of purified SpeB (Toxin Technology, Inc., Sarasota, FL) 3 times at time 0, 6 h, and 12 h. Biofilm was measured at 18 h using CV staining as previously discussed. The level of reduction in biofilm formation was statistically significant ((***) *P *< 0.0001) compared to the untreated samples. MGAS5005Δ*srv*, with constitutive production of SpeB, is presented for comparison.

To prove that SpeB alone is capable of disrupting GAS biofilm formation, we added purified active SpeB (Toxin Technology, Inc., Sarasota, FL)(final concentration 1 μg/mL) 3 times over the course of static biofilm development (0, 6 h, and 12 h). CV staining was performed on treated and untreated samples at 18 h post-seeding (Figure 4). SpeB addition resulted in a significant decrease in measurable biofilm of all treated strains to levels comparable to MGAS5005Δ*srv *(Figure 4).

**Figure 5:**
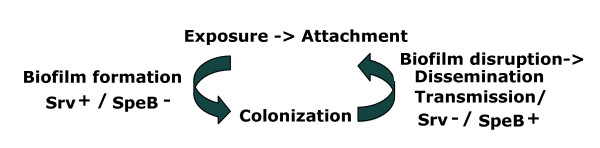
**Hypothetical model of Srv/SpeB mediated GAS biofilm formation and dispersal**. Following GAS exposure, Srv-mediated negative regulation of SpeB production would allow biofilm formation and colonization. As of yet unidentified environmental signals may reverse this control, promoting SpeB production and subsequent biofilm dispersal in order to facilitate dissemination/transmission of the organism. We hypothesize that this cycle is likely held in equilibrium such that controlled amounts of SpeB may be produced to allow dissemination without complete disruption of the GAS biofilm.

Taken together, the data indicate that the biofilm deficient phenotype of MGAS5005Δ*srv *is due to the constitutive production of mature SpeB. Inactivation of *speB *in the MGAS5005Δ*srv *background restored biofilm formation to wild-type levels. Complementation of MGAS5005Δ*srv*Δ*speB *through the addition of exogenous SpeB significantly reduced biofilm formation to MGAS5005Δ*srv *levels. These results support a model in which the Srv mediated control of SpeB production regulates GAS biofilm formation (Figure 5). Following initial exposure and attachment, our model would predict Srv-based negative regulation of SpeB production. This state would allow biofilm formation and colonization. Likewise, an opposite state would be predicted in which SpeB production is upregulated allowing biofilm dispersal and dissemination/transmission of GAS. We hypothesize an equilibrium exists between these two states such that controlled levels of SpeB may be produced to facilitate transmission while preventing complete biofilm disruption. For clarity, it is important to point out that our work was done in the MGAS5005 background, a background which contains a mutation in *covS*, which has been shown to be involved in invasive disease and is characterized by an invasive transcriptome profile[14,15]. Recently, Hollands *et al. *have shown in a separate M1T1 strain (5448) that mutation of *covS *(obtained following passage through an animal model) resulted in a strain with decreased biofilm formation due to increased capsule production[20]. They show that 5448 formed more biofilm than the 5448 *covS *mutant[20]. Thus, our future work is directed at studying the effects of mutation of *srv *in a *covS+ *M1T1 background (as well as in other serotypes) to understand the role of Srv in biofilm formation and GAS disease.

## Competing interests

The authors declare that they have no competing interests.

## Authors' contributions

ALR participated in the design of the study, conducted *in vitro *experiments, and drafted manuscript. RCH designed and developed MGAS5005Δ*srv*Δ*speB *mutant and critically analyzed manuscript. SDR participated in the design of the study and helped to draft the manuscript. All authors read and approved of the final manuscript.
